# The Association between State Attachment Security and State Mindfulness

**DOI:** 10.1371/journal.pone.0116779

**Published:** 2015-03-18

**Authors:** Christopher A. Pepping, Penelope J. Davis, Analise O’Donovan

**Affiliations:** 1 School of Applied Psychology, Griffith University, Brisbane, Australia; 2 Behavioral Basis of Health, Griffith University, Brisbane, Australia; 3 Griffith Health Institute, Griffith University, Gold Coast, Australia; Carnegie Mellon University, UNITED STATES

## Abstract

Recent research suggests that attachment and mindfulness are related, though the nature of this association is unclear. Here we present two studies examining whether there is a causal relationship between state attachment and state mindfulness. Study 1 investigated the effects of experimentally increasing state mindfulness on state attachment security. State mindfulness was successfully enhanced, but this led to no change in state attachment security. Study 2 investigated the effects of experimentally enhancing state attachment security on state mindfulness. State attachment security was successfully enhanced, but this did not lead to any change in state mindfulness. These findings suggest that there is not a direct, immediate causal relationship between state attachment and state mindfulness as a result of brief experimental manipulations. Future research should examine these associations in longer term interventions.

## Introduction

Recent theory and research suggest that attachment and mindfulness are related [1, 2], but the nature of this association remains unclear. Ryan and colleagues [3] propose that attachment and mindfulness may be related bi-directionally, whereby attachment security fosters a greater capacity for mindfulness, and higher levels of mindfulness allow individuals to be less adversely affected by the cognitive and affective responses characteristic of insecure attachment. However, this bi-directional association has yet to be investigated empirically. The present research was designed to do this.

Bowlby [4] proposed that humans have a biologically evolved attachment system that motivates infants to seek proximity to stronger and wiser caregivers to protect the infant from harm. When caregivers are available, sensitive, and responsive, an infant is likely to experience feelings of security, whereas infants who do not experience responsive caregiving, adopt secondary, insecure, attachment strategies [5]. Bowlby [4] proposed that the attachment system is active and influential across the lifespan. Attachment in adulthood is commonly conceptualized and measured along the two dimensions of anxiety and avoidance [6]. Attachment anxiety is characterized by an intense fear of rejection and abandonment, and concern about relationships, whereas attachment avoidance reflects a tendency to feel uncomfortable with intimacy and closeness.

Insecure attachment (high attachment anxiety and/or avoidance) has consistently been shown to predict poor psychosocial adjustment, whereas secure attachment (low anxiety and avoidance) contributes to positive psychosocial outcomes, such as increased well-being and relationship satisfaction, and adaptive emotion regulation [5]. Interestingly, Shaver and colleagues [2] and Ryan and colleagues [3] note that mindfulness is also associated with many of the same outcomes.

Mindfulness is commonly defined as “paying attention in a particular way: on purpose, in the present moment, non-judgementally” [7] (p. 4). Mindfulness is characterised by perceiving thoughts, feelings, and sensations without suppressing or becoming overwhelmed by them [8]. Individual differences exist in ‘naturally occurring’ mindfulness and the capacity for mindfulness [9]. Like secure attachment, high dispositional mindfulness is associated with a wide range of positive psychosocial outcomes [10], which raises the interesting possibility that mindfulness and attachment security are somehow related.

Several studies have demonstrated an association between low attachment anxiety and avoidance with high mindfulness [1, 2, 11, 12]. Pepping and colleagues [1] found that attachment predicted 18.8% of the variance in mindfulness in non-meditators, and 43.3% of the variance in mindfulness for those with a meditation practice, indicating that attachment and mindfulness are related, though to different degrees based on meditation experience. Ryan and colleagues [3] outlined two possible causal pathways between attachment security and mindfulness. First, there may be a common precursor whereby individuals who have experienced sensitive and responsive caregiving in childhood are likely to develop both a secure attachment style and higher levels of mindfulness. Second, there may be a bi-directional causal relationship between the two variables whereby secure attachment fosters increased mindfulness, and individuals who are more mindful may experience greater felt security, and be more open and receptive to relationship partners.

With regards to the bi-directional, causal relationship, individuals with a secure attachment style should be more able to focus attention on the present moment, without worrying about rejection and abandonment (attachment anxiety), or suppressing, avoiding, or defending against threatening experiences (attachment avoidance) [2]. Thus, individuals with a secure attachment style may have greater capacity to be mindful [2, 3]. On the other hand, people who are dispositionally higher in mindfulness may be less likely to be consumed with thoughts and emotions related to insecure attachment [3], such as fear of abandonment and fear of intimacy. Consistent with this possibility is evidence that a three-month full-time intensive Buddhist meditation program led to reductions in attachment anxiety and avoidance [13]. However, the study examined only very experienced meditators (*M* = 13 years of experience), and included loving-kindness and compassion-focussed meditations which overlap considerably with attachment security priming [14, 15]. It is therefore difficult to draw conclusions from this study as to the bi-directional association. Based on these considerations, in the present research we specifically did not use meditations that were similar to security priming (e.g., loving-kindness), and we only included participants who did not have meditation experience.

Theoretically, individuals who are more mindful are also likely to have a secure attachment style [1]. However, the question of whether secure attachment fosters higher levels of mindfulness, and vice-versa, remains unanswered. For the purposes of investigating the association between attachment and mindfulness, we used experimental methods to examine whether inducing a mindful state would lead to enhanced state attachment security (Study 1) and whether priming attachment security would lead to increased state mindfulness (Study 2). To examine these associations experimentally, it was necessary to use state measures of attachment and mindfulness, as one would not expect brief experimental manipulations to alter dispositional attachment or mindfulness. Conceptually, state and dispositional measures have much in common. Dispositional measures capture the frequent occurrence of the state under investigation. In addition, for a state measure to be validated, it is expected that the measure would correlate highly with dispositional measures of the same construct, which further attests to the shared commonality between state and dispositional measures.

If enhancing mindfulness leads to enhanced security, this could inform interventions designed to reduce attachment insecurity. Much is known regarding the antecedents and outcomes of attachment insecurity, but less is known regarding how to enhance attachment security [5]. Study 1 investigated whether increasing mindfulness would increase state attachment security. If increasing mindfulness does indeed lead to increased security, this may suggest that further investigation into the utility of mindfulness based interventions to reduce attachment insecurity is warranted.

If enhancing state attachment security leads to increased state mindfulness, this would be one of the first studies to examine predictors of mindfulness, and may suggest that the origins of mindfulness may be related to attachment. To date, much is known about the benefits of mindfulness. However, very little is known regarding the origins of mindfulness. If increasing attachment security leads to increased state mindfulness, this may suggest that future research should examine attachment as one possible precursor to the development of mindfulness. Study 2 therefore investigated whether enhancing state attachment security would increase state mindfulness.

## Study 1

Study 1 investigated whether increasing state-mindfulness through an experimental mindfulness induction would increase state attachment security, and decrease state attachment anxiety and avoidance. Past research reveals that brief laboratory based mindfulness inductions lead to short-term, immediate effects on psychosocial outcomes. For instance, Broderick [16] induced dysphoric mood in a sample of 177 undergraduate participants, and then randomly assigned them to either a rumination condition, a distraction condition, or a mindfulness meditation condition. Both distraction and mindfulness meditation were more effective in reducing dysphoric mood compared to rumination. However, mindfulness meditation was most effective. Similarly, Arch and Craske [17] examined the effects of a focussed breathing induction (mindfulness of the breath) versus an unfocussed attention condition, and a worry condition, on negative affect and emotional volatility following the viewing of affectively valenced picture slides. Individuals assigned to the mindful breathing condition reported less negative affect and emotional volatility in response to the pictures, and also exhibited greater willingness to view aversive pictures. Brief experimental mindfulness inductions have also been shown to enhance pain tolerance [18] and state self-esteem [19]. Thus, there is clear evidence that experimentally manipulating state mindfulness can lead to a range of theoretically related outcomes, at least in the short term.

If the relationship between attachment and mindfulness exists because higher mindfulness directly leads to greater felt security through adopting a more open, curious and non-judgmental stance to experiences, including relationships, then increasing state mindfulness experimentally should increase state attachment security, and decrease both state attachment anxiety and avoidance. It is important to note that brief experimental manipulations are very unlikely to shift stable working models of relationships. Only long-term interventions are likely to impact on dispositional attachment. Therefore, the present research focussed on state attachment security, known as felt security, which is “a sense that the world is generally safe, that attachment figures are helpful when called upon, and that it is possible to explore the environment curiously and confidently and to engage rewardingly with other people” [5] (p. 21). Evidence indicates that felt security can be experimentally manipulated [5, 14, 15, 20]. Thus, in the present research, we examined whether enhancing state mindfulness would lead to change in state attachment, as this could inform future research investigating more intensive interventions on more stable characteristics. It was predicted that participants in the mindfulness induction condition would show increases in state attachment security, and decreases in state attachment anxiety and avoidance. No such changes were predicted for the control condition.

### Method

#### Participants

Participants were 86 undergraduate psychology students with no mindfulness meditation experience (67 females and 19 males, ranging in age from 16 to 39, *M* = 20.40 years, SD = 4.92), who participated for course credit. This would provide power of. 87 to detect even a small effect (*d* = .3).

#### Measures

As a manipulation check, the five-item Mindful Attention and Awareness Scale-State version (MAAS-State) [9] was administered to assess state mindfulness. The MAAS-State has good internal consistency α = .92, and predicts dispositional mindfulness [9]. Items are reverse scored such that higher scores reflect higher levels of state mindfulness (e.g., “I found it difficult to stay focussed on what was happening in the present” and “I was finding myself preoccupied with the future or the past”). It is generally administered after the completion of a task, and requires participants to reflect on their level of mindfulness during the task. In this research, the reference task was completing the questionnaires prior to this scale. Internal consistency in the present sample was high, α = .86.

The 21-item State Adult Attachment Measure (SAAM) was used to assess changes in state attachment. The SAAM consists of three subscales (anxiety, avoidance and security) [20]. Higher scores on each subscale reflect higher state attachment anxiety (e.g., “I really need to feel loved right now”), security (e.g., “I feel loved”) or avoidance (e.g., “I would be uncomfortable having a good friend or a relationship partner close to me”). The measure has good convergent and discriminant validity [20], and displayed high internal consistency in the present sample α = .84,. 86, and. 93 for anxiety, avoidance and security, respectively.

### Procedure

In both studies, ethics approval was provided by the Griffith University Human Research Ethics Committee, and participants provided written informed consent to participate in the study. There were a small number of participants who were under 18. However, these participants were considered to be able to provide consent to participate without parental consent given their knowledge and maturity being enrolled in a university program, and consent from the next of kin was therefore not obtained. The Griffith University Human Research Ethics Committee approved this protocol. Participants were randomly assigned to either the mindfulness induction condition (*n* = 41) or to the control condition (*n* = 45). Participants were informed that the research was investigating thoughts and feelings about particular experiences. Participants completed a questionnaire package including demographic information, the SAAM and the MAAS-State upon arrival.

In the experimental condition, participants completed a 15-minute mindfulness induction that was read aloud to participants by the experimenter. To ensure that any observed effect of enhancing mindfulness was not specific to any one mindfulness induction, participants completed one of four mindfulness inductions (mindfulness of the breath, thoughts, emotions, and the body). The scripts were adapted from several sources [21, 22], and are available upon request. Participants in the control condition completed one of four tasks that were unrelated to feelings of security or love, and were therefore unlikely to prime attachment security, and were also unlikely to enhance mindfulness (read a 15-minute story about nature, or reflected on their use of listening skills, assertion, or the use of questions in conversation). Following the manipulation, participants again completed the SAAM and MAAS-State.

### Results

A 2 x 2 mixed between-within subjects analysis of variance (ANOVA) was conducted to assess the changes in the MAAS-State as a manipulation check. There was a significant main effect for time for the MAAS-State, *F*(1, 83) = 8.94, *p* = .004, partial η^2^ = .097, and a significant interaction between condition and time (pre-post) *F*(1, 83) = 6.88, *p* = .01, partial η^2^ = .077. MAAS-State score significantly increased between pre (*M* = 20.75) and post (*M* = 23.48) (*t* (39) = -3.87, *p* <. 001) with a moderate effect size (*d* = .56) in the experimental condition, whereas there was no significant difference from pre (*M* = 21.42) to post (*M* = 21.60) (*t* (44) = -.266, *p* = .792) in the control condition (*d* = .04).

With regards to the outcome, three mixed 2 x 2 between-within subjects Analyses of Variance were conducted to assess changes in SAAM anxiety, avoidance and security. For attachment security, there was no significant main effect for time *F*(1, 81) = .543, *p* = .463, partial η^2^ = .007, and no significant interaction between condition and time *F*(1, 81) = .001, *p* = .982, partial η^2^ = .000. For attachment anxiety, there was no significant main effect for time *F*(1, 80) = 1.97, *p* = .164, partial η^2^ = .024, and no significant interaction *F*(1, 80) = 1.102, *p* = .297, partial η^2^ = .014. Finally, for attachment avoidance, there was a significant main effect for time *F*(1, 80) = 4.706, *p* = .033, partial η^2^ = .056, but no significant interaction *F*(1, 80) = .298, *p* = .587, partial η^2^ = .004, indicating that there was change in attachment avoidance in both conditions. However, post-hoc analyses indicated that avoidance did not change in either the control (*p* = .137) or experimental (*p* = .132) condition.

It is important to ensure that these non-significant effects in the outcome are not due to lack of power. The present study had power to detect even a small effect size. Only very small effect sizes were found in the control condition (*d* = .04,. 12,. 06) and experimental condition (*d* = .02,. 09,. 16) for security, anxiety, and avoidance respectively. The lack of change in the outcome is therefore not due to lack of power. In brief, results clearly indicate that enhancing state mindfulness does not lead to a change in state attachment.

### Discussion

In Study 1, state mindfulness increased in the experimental condition but not in the control condition, demonstrating that the manipulation was successful. However, there was no change in state attachment in either condition, indicating that increasing state mindfulness did not lead to changes in state attachment. Several studies have shown that brief mindfulness inductions lead to theoretically related outcomes [23]. The fact that mindfulness increased suggests that participants in the experimental condition were more open and receptive to the present moment, were perceiving their thoughts rather than judging them or avoiding them, were less ruminative, and were more able to ‘step back’ from their thoughts rather than becoming overwhelmed by them. However, these changes in mindfulness abilities did not lead to any increase in felt security. If attachment and mindfulness are related because higher mindfulness directly leads to enhanced security, then increasing state mindfulness should lead to an increase in state-security. This did not occur in Study 1, suggesting that the association between state attachment and state mindfulness may not be because higher state mindfulness allows individuals to be less consumed by attachment insecurities.

However, it is important to note that the measures used tap into fleeting states, and these may or may not reflect more stable trait characteristics. It is possible that the length of the intervention was insufficient to produce change in state attachment, and characteristics of the sample may impact on the generalizability of findings. These points will be discussed further in the general discussion. However, the alternative pathway of the bi-directional association also needs to be investigated: Does increasing attachment security increase mindfulness?

## Study 2

Study 2 investigated whether enhancing state attachment security through established attachment security priming methods would lead to increased state mindfulness. Temporarily activating mental representations of attachment figures (attachment security priming) provides an individual with the symbolic availability of attachment figures, and enhances an individual’s sense of felt security [5, 14]. Mikulincer and Shaver [15] provide an extensive review of numerous security priming techniques which have successfully activated symbolic representations of attachment figures. Numerous studies have found that by activating felt security, individuals (regardless of their attachment style) tend to behave more like those who are *dispositionally* secure [14, 20].

Security enhancement reduces negative reactions to out-group members [14], and leads to greater levels of empathy, as opposed to high levels of personal distress (common in attachment anxiety) or suppression or avoidance of emotional content (common in attachment avoidance) [24]. Security priming has also been shown to lead to increases in self-compassion [25]. Thus, there is clear evidence that enhancing state attachment security leads to a range of theoretically relevant outcomes. If the relationship between attachment and mindfulness exists because individuals with a secure attachment style have a greater capacity for mindfulness, then priming secure attachment should lead to increases in state mindfulness. Specifically, if enhancing state security allows individuals to be temporarily less consumed by cognitive and emotional processes associated with attachment insecurity, they may be temporarily better able to maintain mindful attention. That is, they may be less consumed by thoughts or emotions relating to the past, and better able to focus on what is happening immediately in the present. It was therefore predicted that participants in the security priming condition would show increases in state mindfulness. No such changes were predicted for the control condition.

### Method

#### Participants

Participants were 67 undergraduate psychology students with no experience in mindfulness meditation (48 females and 19 males, ranging in age from 17 to 56, *M* = 21.12 years, SD = 7.32), who participated for course credit. This would provide power of. 81 to detect even a small effect (*d* = .31).

#### Measures

The SAAM [20] was administered as a manipulation check, and had good internal consistency in the present sample, α = .88,. 83, and. 94 for anxiety, avoidance and security respectively. The MAAS-State [9] was used as the outcome measure, and had high internal consistency in the present sample, α = .81.

#### Procedure

Participants were randomly assigned to either the security priming condition (*n* = 33) or to the control condition (*n* = 34). Participants completed a questionnaire package that included the measures mentioned above. In the experimental condition, participants were given one of four 10-minute attachment security primes based on primes used in previous research [14, 24, 26]. In the control condition, participants completed one of four tasks unrelated to attachment and mindfulness (watch a brief clip of waterfalls, or reflect on the use of listening skills, assertion, or the use of questions in conversation). Following the manipulation, participants again completed the SAAM and MAAS-State.

### Results

Three mixed between-within subjects Analyses of Variance were conducted to assess changes in SAAM anxiety, avoidance and security as a manipulation check. For state attachment security, there was a significant main effect for time, *F*(1, 64) = 10.33, *p* = .002, partial η^2^ = .139 and a significant interaction between condition and time *F*(1, 64) = 13.57, *p* <. 001, partial η^2^ = .175. For the experimental condition, there was a significant increase between pre (*M* = 36.76) and post (*M* = 40.76) state attachment security (*t* (32) = -3.867, *p* = .001) with a moderate effect size (*d* = .50), whereas for the control condition, there was no significant difference between pre (*M* = 35.79) and post (*M* = 35.52) scores (*t* (32) = .520, *p* = .607; *d* = .03). No significant effects were found for anxiety or avoidance.

With regards to the outcome, a mixed between-within ANOVA was conducted for the MAAS-State. There was no significant main effect of time, *F*(1, 65) = .027, *p* = .870, partial η^2^ = .000, and no significant interaction between condition and time *F*(1, 65) = .091, *p* = .764, partial η^2^ = .001. Again, the present study had power to detect even a small effect size. Only a very small effect size change was found in the control condition (*d* = .05) and the experimental condition (*d* = .02) for state mindfulness. Results indicate that enhancing state attachment security does not lead to change in state mindfulness.

### Discussion

In Study 2 state attachment security increased in the experimental condition and not in the control condition, indicating that the manipulation was effective. However, this increase in state attachment security did not result in increased state mindfulness. If attachment and mindfulness are related because attachment security directly leads to enhanced mindfulness, then enhancing state attachment security should lead to an increase in state mindfulness. This did not occur in Study 2, suggesting that the association between attachment and mindfulness may not be due to the direct result of increased attachment security providing a greater capacity for mindfulness. Again, it is acknowledged that the manipulation was only a very brief experimental manipulation, and these findings cannot rule out the potential for change in more stable characteristics as a result of longer-term interventions. However, results from Studies 1 and 2 suggest that the relationship between attachment and mindfulness, at least when assessed using state outcomes, may not be a direct, immediate, causal association, for increasing one did not lead to an increase in the other.

## General Discussion

Findings from the present research suggest that there is not a direct, immediate causal association between state attachment security and state mindfulness. Very simply, increasing state security did not lead to increased state mindfulness, nor did increased state mindfulness lead to greater state security. Mindfulness meditation training has consistently led to positive psychological outcomes in both clinical interventions, and when used as an experimental manipulation [23]. In Study 1, evidence that one brief session of mindfulness practice in meditation-naïve students resulted in increased mindfulness but not increased attachment security indicates no *direct* impact of mindfulness on attachment, at least when examined using very brief experimental manipulations. At first glance this finding seems inconsistent with that of Sahdra and colleagues [13]. However, as noted earlier, the sample in the Sahdra and colleagues [13] research was a group of highly experienced meditators, and the intervention was intensive over a three-month period and used meditations that overlapped conceptually with security priming. It is therefore difficult to compare the present results with this study.

In Study 2, security priming led to increased state attachment security, but not increased state mindfulness, suggesting that the relationship between attachment and mindfulness may not be due to the *direct* impact of state attachment security on mindfulness. The lack of change in the outcomes in both studies is not due to lack of power. Both studies had adequate power to detect small to moderate effects. The very small effect sizes observed in the outcomes (security and mindfulness; *d* = .02 for each) are too small to be of theoretical or practical importance. It is important to note that in both studies, the specific experimental tasks (i.e., priming or mindfulness induction) were pre-selected and alternated between sessions so that any effects were not simply due to the specific prime or mindfulness induction that was used. The manipulations were effective in both studies. However, these changes in mindfulness (Study 1) and attachment security (Study 2) did not lead to change in the outcome. A strength of this research is that this lack of change in the respective outcome is unlikely to be due to the lack of effectiveness of one specific prime or mindfulness induction.

Findings of the present research suggest that the relationship between attachment and mindfulness may not be a direct, immediate causal association. Instead, the relationship may be more distal or indirect, and longer-term interventions may be necessary to observe effects. It is also possible that the two variables may be related because they share a common developmental precursor. Much is known about factors that impact on attachment, such as sensitive and responsive caregiving received in childhood [5, 27], but remarkably little is known about factors that impact on mindfulness. Perhaps such sensitive and responsive caregiving fosters not only the development of a secure attachment style, but other positive psychosocial outcomes, including mindfulness.

Ryan and colleagues [3] proposed that individuals who receive sensitive and responsive caregiving that is characterised by love and support for autonomy may be more likely to display higher levels of dispositional mindfulness. Specifically, the development of mindful awareness and capacity for self-observation is “facilitated by providers who can be attuned to, mirror, and resonate with the infant’s experience” [3] (p. 180), whereas individuals who grow up in abusive or rejecting environments may have this capacity for mindful attention impaired [3]. To date, only one study has investigated this proposition empirically. Walsh and colleagues [28] investigated whether parental nurturance predicted individual differences in dispositional mindfulness. Contrary to the authors’ predictions, parental nurturance did not predict mindfulness. However, participants’ experience with mindfulness meditation practice was not assessed, and it is therefore difficult to draw firm conclusions from this study. Specifically, given that experience in mindfulness meditation leads to increases in mindfulness [29], in order to accurately examine predictors of ‘naturally occurring’ dispositional mindfulness, it is necessary to exclude individuals who have engaged in practices that are designed to enhance mindfulness. Given that individuals who have received low parental nurturance during childhood experience cognitive and emotional difficulties [30, 31, 32], it is possible that these difficulties may prompt individuals to pursue mindfulness meditation. However, future research needs to test this proposition empirically.

In addition, the relationship between parenting received in childhood and dispositional mindfulness is likely to be complex, and may be indirect. It is possible that parenting received in childhood that is characterised by sensitivity and warmth may facilitate the development of healthy emotion regulation abilities, which may in turn provide an individual with an enhanced capacity for mindfulness. Much evidence converges to reveal that sensitive and responsive caregiving received in childhood leads to the development of adaptive self-soothing and emotion regulation abilities [30, 31, 32, 33], and these emotion regulation abilities are also associated with both mindfulness [34, 35] and attachment [5, 36]. Thus, it seems plausible that sensitive and responsive caregiving may foster not only secure attachment, but also an enhanced capacity for mindfulness by providing an individual with the necessary self-soothing and emotion regulation abilities. Future research should examine the origins of individual differences in dispositional mindfulness.

As mentioned earlier, it is also possible that the brief nature of the experimental manipulations, although successful at increasing state mindfulness (Study 1) and state attachment security (Study 2), was not sufficient to produce change in the outcome, even when assessed as state variables. Recent evidence indicates that the association between attachment and mindfulness is mediated by emotion regulation processes [11, 37]. Pepping and colleagues [37] found that attachment anxiety and avoidance each predicted low mindfulness, and these associations were fully mediated by difficulties in emotion regulation. Similarly, Caldwell and Shaver [11] found that attachment anxiety predicted low mindfulness, and this association was mediated by rumination and poor attentional control. Attachment avoidance was also associated with low mindfulness, and this association was mediated by thought suppression and poor attentional control. Thus, there is emerging evidence that the association between attachment and mindfulness is indirect. Given this, it is possible that the very brief experimental manipulations in the present research were not sufficient to produce change in the emotion regulation processes that mediate the attachment-mindfulness association, and thus no change in the respective outcome was observed.

It is possible that longer-term mindfulness interventions may lead to change in attachment over time via a state-by-trait process. Specifically, it is possible that, over time, frequent experiences of a mindful state might begin to shape greater trait mindfulness and the associated emotion regulation capacity, which may in turn enhance security. Findings reported by Sahdra and colleagues [13] are certainly consistent with this proposition. However, as mentioned earlier, the nature of the sample and types of meditations limits the extent to which conclusions can be made. The type of mindfulness intervention described by Caldwell and Shaver [38] for women who had experienced unhealthy attachment relationships resulted in decreased rumination and suppression, and increased emotion regulation. Such interventions may, over time, reduce the cognitive and emotional processes associated with attachment insecurity, and thus lead to increased security. Further research exploring the utility and efficacy of long-term mindfulness interventions on attachment security is greatly needed to examine the possibility of state interventions leading to long-term changes in traits. It is also possible that, over time, attachment security may lead to greater mindfulness. Only longitudinal research can definitively address this proposition, but theoretically it seems likely. Such research would also illuminate the potential origins of individual differences in dispositional mindfulness.

### Conclusions, Limitations & Future Directions

Limitations of the present research need to be acknowledged. Firstly, although attachment security increased in the experimental condition in Study 2, attachment anxiety and avoidance did not decrease. Although based on prior research, increasing attachment security should lead to change in state attachment anxiety and avoidance [20], perhaps directly targeting only security was not sufficient to produce change in state anxiety and avoidance with the specific primes used here. This was unexpected, as trait measures of attachment security generally conceptualize attachment security as low attachment anxiety and avoidance [5, 6]. However, in the development and validation of the State Adult Attachment Measure, Gillath and colleagues [20] found that, at least for the purposes of assessing state attachment, security, anxiety, and avoidance emerged as three distinct factors. In the present research, it is possible that if attachment anxiety and avoidance had decreased, changes in mindfulness might have been observed. However, this possibility seems unlikely. Increased security in the experimental condition suggests that individuals experienced a greater sense of felt security. If the association between attachment and mindfulness exists because attachment security directly enhances mindfulness, the increase in felt security should have led to higher levels of state mindfulness in the experimental condition, and not in the control group where state attachment security remained unchanged.

Although findings from the experimental studies show no evidence of a direct, immediate causal association, the possibility remains that extended mindfulness training may, over time, reduce the emotion regulation difficulties that characterise insecure attachment, and in turn, enhance attachment security. Further research exploring the utility and efficacy of long-term mindfulness interventions on attachment security is greatly needed. It is important to note that in both studies, participants were not explicitly asked the degree to which they engaged with the various tasks within the experimental session. Although the manipulations were effective as assessed by the manipulation checks, the fact that individual assessment of engagement with the tasks was not administered is a limitation that should be kept in mind when interpreting the results.

Finally, it is important to consider the influence of the sample characteristics on results of the present research. Although the inclusion of only individuals with no prior mindfulness experience was a deliberate component of the study design for reasons mentioned earlier, the fact remains that the sample was a relatively homogenous group of young, undergraduate students who were participating for experimental credit rather than intrinsic reasons. The possibility remains that results could be different using a different study sample. This should be addressed in future research.

Findings from the present research suggest that the association between mindfulness and attachment is not a direct, immediate causal association, for increasing one via brief experimental manipulations did not lead to an increase in the other. Future research is needed to examine longer-term interventions on more stable characteristics, and also to examine the origins of individual differences in dispositional mindfulness.

## Supporting Information

S1 Data Data file for Study 1 (SAV). (SAV)

S2 Data Data file for Study 2 (SAV). (SAV)
